# Where to for Sexual Health Education for Adolescents in Sub-Saharan Africa?

**DOI:** 10.1371/journal.pmed.1000288

**Published:** 2010-06-08

**Authors:** Rachel Jewkes

**Affiliations:** Gender & Health Research Unit, Medical Research Council, Pretoria, South Africa

## Abstract

Rachel Jewkes discusses disappointing results from a school-based sexual health intervention study in Tanzania and their implications for future health education programs.

**Linked Research Article**

This Perspective discusses the following new study published in *PLoS Medicine*:

Doyle AM, Ross DA, Maganja K, Baisley K, Masesa C, et al. (2010) Long-Term Biological and Behavioural Impact of an Adolescent Sexual Health Intervention in Tanzania: Follow-Up Survey of the Community Based MEMA kwa Vijana Trial. PLoS Med 7(6): e1000287. doi:10.1371/journal.pmed.1000287.

David Ross and colleagues conduct a follow-up survey of the community-based MEMA kwa Vijana (“Good things for young people”) trial in rural Tanzania to assess the long-term behavioral and biological impact of an adolescent sexual health intervention.

## Introduction

Sub-Saharan Africa remains the global hub of the HIV epidemic. Seventy percent of new HIV infections occur in the region. And because the sexually transmitted epidemic starts among women and men in their teenage years [1], promoting the sexual health of adolescents is a substantial health priority. In light of this, the findings of the MEMA kwa Vijana trial in Tanzania [2] and the long-term follow up of participants in its school-based intervention, published this week in *PLoS Medicine* [3], are very disappointing. The key questions we have to ask now are: In light of current knowledge about behaviour change, are these findings surprising? And, what are the implications for the next generation of sexual health interventions?

In the 15 years since the process of developing the MEMA kwa Vijana intervention began, understanding of adolescent behaviour change has deepened (not least from this study) to the point where it seems less than surprising that this intervention was unsuccessful. At the time of its design, a major concern was that good behaviour change intentions would be undermined by poor services from clinics and untreated sexually transmitted infections. However, it now seems that the biggest threat to such interventions comes from the context of delivery in the school environment and the constraints thus entailed.

Social science research from the MEMA kwa Vijana team now indicates that the school-based intervention was being implemented in a context of massive gender and status power differentials between teachers and learners, which provided the opportunity for rape, harassment, economic exploitation, and beating of learners, thus severely undermining positive messages from the programme [4]. Broader community engagement with the programme was very limited and, because it was delivered in schools, it had to be tailored to the constraints of the classroom. As a result, the curriculum was delivered in 12 lessons per year of 40 minutes each, for a maximum of 3 years [3]. In practice, for many this was probably much less than 8 hours of intervention, and was at most 24 hours for the two-thirds of learners who attended all lessons over all 3 years [3]. This intervention was thus low intensity, particularly if compared, for example, to the 50 hours of an intervention like Stepping Stones (the author adapted Stepping Stones for South Africa) [5] or the 5-day Men As Partners program [6]. Furthermore, the Department of Education in Tanzania prohibited condom promotion and demonstrations in school and demanded a focus on abstinence, a strategy that has been shown to be ineffective [7]. Teachers had only short training in delivering the intervention, and since they were unfamiliar with participatory methods, it was very substantially didactic [8]. There were also some notable omissions in the curriculum. It lacked a focus on skills building, particularly communication skills, which are recognised now as essential for good practice [8],[9]. It was also notably limited in addressing gender relations and identities.

## The Challenges of Sexual Health Interventions

In their paper reporting the impact of the intervention 9 years after it was implemented, David Ross et al. [3] note that this intervention, although remarkable for the extent and rigor of its evaluation, now joins the ranks of a much larger set of school-based interventions in sub-Saharan Africa that have been shown to be ineffective in changing sexually risky behaviour. Plummer et al. [4] present a stark picture of the school environment that clearly shows how critically important context is for the success of HIV prevention interventions. This is echoed by a growing body of educational theory that seeks to explain why many HIV interventions in schools have been ineffective, and it points to the importance of transforming schools, and educational policy, as a whole. An important contribution here has been made by Unterhalter [10] and elaborated in Morrell et al. [11]. After a decade of empirical research on gender and HIV in schools in South Africa, they reflect that policy approaches to gender, violence, and HIV have three dimensions, which at best hold the potential for being profoundly empowering for learners. These are: *interventions*, which focus, for example, on introducing ideas, giving information, providing skills, or punishing transgressive behaviours; *institutions*, with their policies able to shape social relations and ways of working; *interactions*, which involve all social relationships in schools, and provide the daily space in which gender identities and relations are enacted and where values are conveyed and may be contested. Unterhalter and Morrell et al. argue that empowerment requires good practice in all three of these dimensions, yet there has been an overwhelming focus on some interventions, more limited attention to institutions through laws and policies and their implementation, and a substantial neglect of attention to interactions. This narrows the frame of many interventions, and may explain the apparent ineffectiveness.

## Where To from Here?

The findings of Ross et al. [3] should not be interpreted as showing that school-based health education programmes in sub-Saharan Africa do not work, but rather that we must learn to do them better. We do not know yet about how to deliver effective HIV prevention through schools. This study reminds us that we cannot safely assume that evidence of attitude change and some behaviour change found in evaluations will translate into long-term protection against HIV or other sexually transmitted infections. If we are to develop an evidence base for programming in schools, substantial resources are needed for evaluations of promising programmes against robust outcomes. We need more research, more large studies, and much more resources.

A central part of this agenda must involve strengthening schools. Ross et al.'s study highlights huge problems in Tanzanian schools that ultimately impact on national development and achievement of the Millenium Development Goals. High quality schooling is critical for development. Schools have an unparalleled potential for reaching a large and critical sector of the population with ideas and skills. The changes that are needed in the school environment to make schools appropriate and effective places for changing sexual practices and gender norms are largely the same needed to provide effective education for development. The opportunity to unite HIV prevention research to these broader agendas should not be missed.

Until we develop the knowledge base for work in schools, much more emphasis needs to be given to scaling up delivery of HIV prevention interventions to youth in contexts where the curriculum content, skills emphasis, duration, selection of facilitator, use of participatory methods, and broader context can be controlled. There are several such programmes in use in sub-Saharan Africa. One is the Stepping Stones programme, which remains the only intervention in sub-Saharan Africa that has been shown to have an impact on a biological indicator of sexual risk [5]. The secret to its success seems to lie in the fact that it was a participatory, gender-transformative intervention that heavily emphasised skills building, both critical reflection—in other words, it taught and emphasised thinking skills—as well as communication skills, rather than focusing on knowledge. There is an urgent need for a dual strategy of investing in further large-scale, rigorous intervention research and in the interim rolling out best practice.
